# Comparative analysis of different response criteria at early phase after PD-1 blockade in non-small lung cancer

**DOI:** 10.1186/s40644-023-00538-x

**Published:** 2023-03-01

**Authors:** Kyoichi Kaira, Ou Yamaguchi, Ichiro Naruse, Yukihiro Umeda, Takeshi Honda, Satoshi Watanabe, Kosuke Ichikawa, Shin Yanagisawa, Norimitsu Kasahara, Tetsuya Higuchi, Kosuke Hashimoto, Yu Miura, Ayako Shiono, Atsuto Mouri, Hisao Imai, Kunihiko Iizuka, Tamotsu Ishizuka, Koichi Minato, Satoshi Suda, Hiroshi Kagamu, Keita Mori, Nobuhiko Seki, Ichiei Kuji

**Affiliations:** 1 Department of Respiratory Medicine, International Medical Center, Saitama Medical University, 1397-1 Yamane, Hidaka-City, Saitama 350-1298 Japan; 2 Department of Respiratory Medicine, Hidaka Hospital, 886, Nakao-cho, Takasaki, 370-0001 Japan; 3 Third Department of Internal Medicine, Faculty of Medical Sciences, University of Fukui, 23-3, Matsuoka-Shimoaizuki, Eiheiji, Fukui 910-1193 Japan; 4 Division of Medical Oncology, Department of Internal Medicine, Teikyo University School of Medicine, 2-11-1, Kaga, Itabashi-ku, Tokyo, 173-8606 Japan; 5 Department of Respiratory Medicine and Infectious Diseases, Niigata University Graduate School of Medical and Dental Sciences, 1-757 Asahimachidori, Chuouku, Niigata, 951-8510 Japan; 6 Department of Radiology, Shinshu University School of Medicine, 1-1-3, Asahi, Matsumoto-City, Nagano 390-8621 Japan; 7 Innovative Medical Research Center, Gunma University Hospital, Showa-machi, 3-39-15, Maebashi, Gunma 371-8511 Japan; 8 Department of Diagnostic Radiology and Nuclear Medicine, Gunma University Hospital, Showa-machi, 3-39-15, Maebashi, Gunma 371-8511 Japan; 9 Department of Internal Medicine, Public Tomioka General Hospital, 1-2073, Tomioka, Gunma 370-2316 Japan; 10 Division of Respiratory Medicine, Gunma Prefectural Cancer Center, 617-1, Takabayashinishi-cho, Ota, Gunma 373-8550 Japan; 11 Cancer Center, Hidaka Hospital, 886, Nakao-cho, Takasaki, 370-0001 Japan; 12 Clinical Research Center, Shizuoka Cancer Center, 1007, Shimonagakubo, Sunto-gun, Shizuoka 411-8777 Japan; 13 Department of Nuclear Medicine, International Medical Center, Saitama Medical University, 1397-1 Yamane, Hidaka-City, Saitama 350-1298 Japan

**Keywords:** ^18^F-FDG PET, PD-1 blockade, Immunotherapy, Lung cancer, Early response, PET response criteria

## Abstract

**Purpose:**

To compare different response criteria using computed tomography (CT) and positron emission tomography (PET) in measuring response and survival in the early phase after programmed death-1 (PD-1) blockade monotherapy in patients with advanced non-small cell lung cancer (NSCLC).

**Methods:**

A total of 54 patients with advanced NSCLC who had 2-deoxy-2-[fluorine-18]-fluoro-D-glucose PET or CT at baseline, and 4 and 9 weeks after PD-1 blockade, were registered. Therapeutic response was assessed according to the Response Evaluation Criteria in Solid Tumors (RECIST), the immune-modified RECIST (irRECIST), the PET Response Criteria in Solid Tumors (PERCIST), the immune-modified PERCIST (iPERCIST), and the European Organization for Research and Treatment of Cancer (EORTC) criteria for dichotomous groups, such as responders vs. non-responders and controlled vs. uncontrolled diseases. Cohen’s κ was used to evaluate the concordance among the different criteria.

**Results:**

The concordance between CT and PET response criteria was fair or slight for responders vs. non-responders, but the agreement between iPERCIST and irRECIST was moderate for controlled vs. uncontrolled diseases. The agreement between EORTC and PERCIST or iPERCIST in detecting responders was higher in the application of metabolic tumor volume (MTV) and total lesion glycolysis (TLG) than in the standardized uptake value corrected for lean body mass (SUL)_peak_. To distinguish controlled from uncontrolled disease, RECIST, irRECIST, and PET criteria (PERCIST, iPERCIST, and EORTC) defined by MTV or TLG were found to be significant predictors of progression-free survival. To distinguish responders from non-responders, iPERCIST by SUL_peak_ or EORTC by TLG were identified as significant indicators. The EORTC criteria using TLG for the detection of responders or uncontrolled diseases had a significantly higher predictive value for response assessment.

**Conclusions:**

The EORTC criteria based on TLG for the early detection of responders and uncontrolled disease were effective as a response assessment at 4 weeks after the PD-1 blockade. When SUL_peak_ was not used but MTV or TLG was, the agreement between EORTC and PERCIST or iPERCIST was almost perfect.

**Supplementary Information:**

The online version contains supplementary material available at 10.1186/s40644-023-00538-x.

## Introduction

Immunotherapy is widely administered to patients with various types of neoplasms. In particular, immune checkpoint inhibitors (ICIs), such as anti-programmed death-1 (PD-1) or PD-ligand-1(PD-L1) antibodies, greatly affect survival in patients with advanced non-small cell lung cancer (NSCLC) [1, 2]. Even though many investigations have attempted to identify an optimal predictor of PD-1 blockade, the confirmation of established biomarkers has failed, aside from the expression of PD-L1 within tumor cells [3]. Therefore, we prospectively examined the prognostic significance of metabolic activity to determine whether 2-deoxy-2-[fluorine-18]-fluoro-d-glucose (^18^F-FDG) positron emission tomography (PET) can predict the efficacy of PD-1 blockade in the early phase after its initiation [4]. It was confirmed that ^18^F-FDG PET was superior to computed tomography (CT) for the detection of responders and non-responders 1 month after PD-1 blockade administration [4]. In our previous study, the PET Response Criteria in Solid Tumors (PERCIST) was adopted as a therapeutic response criterion for ^18^F-FDG PET [5]. But it is still unclear which response criteria are best for distinguishing responders from non-responders in the early phase after PD-1 blockade therapy.

Nowadays, tyrosine kinase inhibitors (TKIs) targeting the genomic alternations such as epidermal growth factor receptor (*EGFR*) mutation, anaplastic lymphoma kinase (*ALK*)-rearrangement and Kirsten Rat Sarcoma Viral Oncogene Homolog (*KRAS*) mutations are currently in clinical development and are proven to markedly improve survival in lung cancer [6]. In patients without these oncogenic alternations, immunotherapy or cytotoxic agents including platinum-based regimens are identified as standard care for systemic treatment [7–9]. However, overcoming the resistance of oncogenic alternations is a critical issue and many challenges have been previously tried to elucidate the mechanism of resistance [6]. Although the mechanism of resistance to immunotherapy also remains unclear, it is necessary to detect the progressive disease at early phase as possible after immunotherapy initiation. Therefore, we should develop an appropriate criteria for evaluating the therapeutic response after immunotherapy administration.

Recently, Beer et al. compared the Response Evaluation Criteria in Solid Tumors (RECIST) 1.1, immune-modified RECIST (irRECIST) [10], and PERCIST 1.0 for the response evaluation of PD-1 blockade in 42 patients with NSCLC after 2 months of therapy [11]. Their study showed moderate agreement between metabolic and anatomic response criteria; however, no significant difference was found in their ability to predict the outcomes [11]. Castello et al. compared RECIST, irRECIST, PERCIST, the European Organization for Research and Treatment of Cancer (EORTC) criteria, and the immune-modified PERCIST (iPERCIST) [12] for the prediction of therapeutic response and outcome at three or four cycles after PD-1 blockade in 52 patients with advanced NSCLC, and the iPERCIST criteria could correctly predict the therapeutic response and survival compared to the other criteria [13]. Other studies have also described that the RECIST, iPERCIST, and EORTC criteria after four to eight cycles of PD-1 blockade could accurately reflect the tumor response and predict the outcome in patients with advanced NSCLC [14, 15]. However, there is still no information regarding the optimal response criteria 1 month after PD-1 blockade treatment. The accurate prediction of immunotherapy at an early phase can prevent the delay of sequential therapy and contribute to prolonged survival.

Based on this background, we compared RECIST, irRECIST, PERCIST, iPERCIST, and EORTC criteria for the evaluation of therapeutic response and outcome in the early phase (4 weeks) after PD-1 blockade monotherapy, using a sample from our previous study [4]. As a result, we assessed which response criteria can be used to predict therapeutic efficacy within 1 month of ICI initiation.

## Methods

### Patients and study design

The present study design has been previously reported [4]. This study was a multicenter prospective investigation in which eight Japanese institutions participated. Patients who had ^18^F-FDG PET or CT at baseline and 4 to 9 weeks after PD-1 blockade monotherapy were included in this study. A total of 54 patients with advanced NSCLC (38 males and 26 females; median age, 73 years; age range, 42–84 years) who received pembrolizumab, nivolumab, and atezolizumab as first- or second-line therapy or further were registered between January 2019 and October 2020. Clinical data were extracted from medical records, and the sample of this study overlapped with that of our previous study [4]. This study was approved by the Institutional Review Board (Saitama Medical University) and conducted according to the Declaration of Helsinki. All patients provided written informed consent before participation and could withdraw from the study at any time. This trial was registered in the Japan Registry of Clinical Trials (registration no. jRCTs031180036; dated: 01/11/2018).

### PET imaging and data analysis

The performance and data analysis of PET imaging have been previously described [4]. The patients fasted for at least 6 h before ^18^F-FDG administration for PET, which was performed using a PET or CT scanner. Three-dimensional data acquisition was initiated 60 min after the FDG injection. Eight bed positions were acquired based on the imaging range. Attenuation-corrected transverse images obtained with ^18^F-FDG were reconstructed with the ordered-subset expectation-maximization algorithm based on the point spread function into 168 × 168 matrices with a slice thickness of 2 mm. For semi-quantitative analysis, the standardized uptake value (SUV) in lean body mass (LBM) corrected for the Japanese population was obtained based on the injected dosage of ^18^F-FDG, the patient’s body weight, and the cross-calibration factor between PET and the dose calibrator. The SUV correction by LBM was based on the literature.^24^ The SUV corrected for LBM (SUL) and Japanese-corrected LBM (JLBM) are defined as follows:

$$\textrm{SUL}=\textrm{radioactive}\ \textrm{concentration}\ \textrm{in}\ \textrm{the}\ \textrm{volume}\ \textrm{of}\ \textrm{interest}\ \left(\textrm{VOI}\right)\ \left(\textrm{MBq}/\textrm{g}\right)/\textrm{injected}\ \textrm{dose}\ \left(\textrm{MBq}\right)/{\textrm{patient}}^{'}\textrm{s}\ \textrm{LBM}\ \left(\textrm{g}\right).$$

$$\textrm{JLBM}\ \textrm{in}\ \textrm{males}=28.27\times \textrm{height}\ \left(\textrm{m}\right)+0.359\times \textrm{weight}\ \left(\textrm{kg}\right)-0.032\times \textrm{age}\ \left(\textrm{years}\right)-21.83$$

$$\textrm{JLBM}\ \textrm{in}\ \textrm{females}=26.12\times \textrm{height}\ \left(\textrm{m}\right)+0.253\times \textrm{weight}\ \left(\textrm{kg}\right)-0.022\times \textrm{age}\ \left(\textrm{years}\right)-19.58$$

CT for initial staging was performed with an intravenous contrast medium, and board-certified radiologists interpreted the images. We used the RAVAT software (Nihon Medi-Physics Co., Ltd., Japan) on a Windows workstation to calculate the maximum SUL (SUL_max_), peak SUV normalized by LBM (SUL_peak_), metabolic tumor volume (MTV), and total lesion glycolysis (TLG) in a semi-automatic manner. TLG is defined as MTV multiplied by the SUL_mean_, of each lesion using the SUL thresholds in the liver VOI. The average of 1.5 × SUL (SUL_mean_) plus 2 × standard deviations of SUL in the liver was used to define each threshold. These SUL thresholds were the optimum values for generating a three-dimensional VOI in which the whole tumor mass was completely enclosed in all cases, using the CT image as the reference. Regions of activity other than tumors, including the myocardium, gastrointestinal tract, kidneys, and urinary tract, were eliminated manually according to the orientation provided by the board-certified nuclear medicine physician. In this study, SULs between facilities and devices were not harmonized.

### Different response criteria

Based on RECIST 1.1, irRECIST, PERCIST, iPERCIST, and EORTC criteria, the response of the tumor was confirmed [5, 16–18]. Details of these guidelines are presented in Table 1. The tumor response by RECIST 1.1 was classified as a complete response (CR), a partial response (PR), a stable disease (SD), and a progressive disease (PD) [19]. For irRECIST criteria, the definition of CR, PR, and SD was the same as that of RECIST, but confirmed PD was used, unlike unconfirmed PD in RECIST. PD by irRECIST should be confirmed by a follow-up examination at 4 to 9 weeks [18].

**Table 1:** PET and CT-based response evaluation criteria for immunotherapy

^**18**^F-FDG PET-based criteria				CT-based criteria		
Response	PERCIST	iPERCIST	EORTC	Response	RECIST 1.1	iRECIST
**CMR**	Complete resolution of ^18^F-FDG uptake within TLs and disappearance of all other lesions to background	As per PERCIST	As per RECIST 1.1	**CR**	Disappearance of all TLs and NTLs, all LNs < 10 mm in short-axis diameter	As per RECIST 1.1
**PMR**	> 30% relative reduction and > 0.8 absolute decrease in SUL_peak_ of hottest lesion	Reduction in sum of SUL_peak_ by at least 30%	After 1 cycle; > 15% of reduction After ≥2 cycle; > 25% of reduction	**PR**	> 30% decrease in SOM, no new lesions	As per RECIST 1.1
**SMD**	Neither CMR, PMR, nor PMD	As per PERCIST	Neither CMR, PMR, nor PMD	**SD**	Neither CR, PR, nor PD	As per RECIST 1.1
**PMD**	> 30% relative increase and > 0.8 absolute increase in SUL_peak_ of hottest lesions and unequivocal progression of ^18^F-FDG uptake NTLs or new ^18^F-FDG uptake lesion	Unconfirmed PMD of > 30% relative increase needs to be confirmed by > 30% increase at 4 weeks later	> 25% increase of SUV	**PD**	> 20% increase in SOM of TLs or unequivocal progression of NTLs, or appearance of new lesion	PD by RECIST 1.1 in iUPD needs to be confirmed by follow-up examination of 4-8 weeks

*Abbreviations*: *TLs* Target lesions, *NTLs* Nontarget lesions, *SOM* Sum of diameter, *LN* Lymph node, *CMR* Complete metabolic response, *PMR* Partial metabolic response, *SMD* Stable metabolic disease, *PMD* Progressive metabolic disease, *CR* Complete response, *PR* Partial response, *SD* Stable disease, *PD* Progressive disease, *iUPD* Unconfirmed PD

PERCIST was classified as complete metabolic response (CMR), partial metabolic response (PMR), stable metabolic disease (SMD), and progressive metabolic disease (PMD) according to changes in SUL_peak_ [14]. For iPERCIST, unconfirmed PMD needs to be confirmed by more than a 30% increase 4 weeks later [17]. The definition of the EORTC criteria is based on the change in SUV, which is different from that of RERCIST [16]. In the current study, in addition to SUL_peak_ and SUV, MTV and TLG also used the PERCIST, iPERCIST, and EORTC criteria.

As an indicator of response assessment, all patients were classified as responders (CR or CMR and PR or PMR) or non-responders (SD or SMD and PD or PMD) and as having controlled (CR or CMR, PR or PMR, and SD or SMD) or uncontrolled (PD or PMD) disease [12, 14].

### Statistical analysis

Statistical analyses were performed using the student’s *t-*test and χ^2^ test for continuous and categorical variables, respectively. The statistical significance level was set at *p* < 0.05. Univariate and multivariate analyses of the relationship between scoring by ^18^F-FDG uptake and different variables were performed using logistic regression. Progression-free survival (PFS) was defined as the time from the initial immunotherapy to disease progression or death, and overall survival (OS) was defined as the time from the initial immunotherapy to death from any cause. The Kaplan-Meier method was used to estimate survival as a function of time, and survival differences were analyzed using the log-rank test. The metabolic responses at 4 and 9 weeks after PD-1 blockade injection were evaluated according to different response criteria on PET [4]. Concordance was evaluated using Cohen’s κ coefficient. Agreement between the two assessments was categorized as poor (weighted κ = 0), slight (weighted κ = 0-0.20), fair (weighted κ = 0.21-0.40), moderate (weighted κ = 0.41-0.60), substantial (weighted κ = 0.61-0.80) and almost perfect (weighted κ > 0.80) [7].

All statistical analyses were performed using GraphPad Prism (v.8.0; GraphPad Software, San Diego, CA, USA) and JMP 14.0 (SAS Institute Inc., Cary, North Carolina, USA).

## Results

### Patient’s demographics

The characteristics of the 54 patients were previously described [4]. Briefly, the performance status (PS) of 0, 1, and 2 was observed in 12, 31, and 11 patients, respectively, and the histology of adenocarcinoma, squamous cell carcinoma, and others were found in 29, 15, and 10 patients, respectively. Forty-two (78%) patients had a history of smoking, and 44 (81%) patients had no driver mutations. PD-L1 expression of < 1%, 1–49, > 50%, and unknown was observed in 14, 12, 19, and 19 patients, respectively. Twenty-two patients were treated with PD-1 blockade as a first-line treatment, and 32 patients were treated with second- or later-line treatments. For PD-1 blockade, nivolumab, pembrolizumab, and atezolizumab were administered to 25, 28, and one patient, respectively. The median follow-up period was 289 days (range: 75–741 days).

### The concordance rate of different response criteria

The objective response rate and disease control rate were 35 and 65%, respectively (19 patients with PR, 16 with SD, and 19 with PD). Of the 54 patients, two (3.7%) experienced pseudo-progression.

A tumor response assessment according to responder or non-responder status between the CT and PET criteria was performed. There was a fair agreement (κ = 0.22-0.30) between PERCIST or iPERCIST and RECIST or irRECIST, whereas there was a slight agreement (κ = 0.10 and 0.11) between EORTC and RECIST or irRECIST (Table 2). When analyzing the PET response criteria by MTV instead of SUL_peak_, a slight agreement (κ = 0.18) was observed between irRECIST and PERCIST; however, a fair agreement (κ = 0.21 ~ 0.33) was identified among the other criteria (Table 2). In the TLG analysis, a fair agreement (κ = 0.28) was observed between iPERCIST and RECIST, and a slight agreement (κ = 0.15 ~ 0.18) was observed among the other criteria (Table 2). Moreover, the tumor response assessment based on responders or non-responders was compared between the different PET criteria (Table 3). A substantial agreement (κ = 0.71 and 0.62) was observed between EORTC and PERCIST or iPERCIST, whereas the analysis of response criteria by MTV and TLG instead of SUL_peak_ depicted almost perfect agreement (κ = 0.89 and 0.91) between EORTC and PERCIST or iPERCIST (Table 3). The agreement between RECIST and irRECIST was almost perfect (κ = 1.0) (Table A1, available online only). Even though there was a substantial agreement (κ = 0.74) between PERCIST and iPERCIST, an almost perfect agreement (κ = 1.0) was observed in the analysis of MTV and TLG (Table A2, available online only).

**Table 2:** Comparison of therapeutic response evaluation between CT and PET criteria

Variables	RECIST 1.0				irRECIST			
	Responder	Non-responder	Total	(κ)	Responder	Non-responder	Total	(κ)
**PERCIST**								
**SUL**_**peak**_								
**Responder**	4	15	19	(κ = 0.22)	4	15	19	(κ = 0.22)
**Non-responder**	1	34	35		1	34	35	
**Total**	5	49	54		5	49	54	
**MTV**								
**Responder**	3	9	12	(κ = 0.25)	3	9	12	(κ = 0.18)
**Non-responder**	2	40	42		2	40	42	
**Total**	5	40	54		5	49	54	
**TLG**								
**Responder**	3	12	15	(κ = 0.18)	3	12	15	(κ = 0.18)
**Non-responder**	2	37	39		2	37	39	
**Total**	5	49	54		5	49	54	
**iPERCIST**								
**SUL**_**peak**_								
**Responder**	4	12	16	(κ = 0.28)	4	11	15	(κ = 0.30)
**Non-responder**	1	37	38		1	38	39	
**Total**	5	49	54		5	49	54	
**MTV**								
**Responder**	4	10	14	(κ = 0.33)	3	9	12	(κ = 0.25)
**Non-responder**	1	39	40		2	40	42	
**Total**	5	49	54		5	49	54	
**TLG**								
**Responder**	3	12	15	(κ = 0.28)	3	12	15	(κ = 0.18)
**Non-responder**	2	37	39		2	37	39	
**Total**	5	49	54		5	49	54	
**EORTC criteria**								
**SUL**_**peak**_								
**Responder**	3	17	20	(κ = 0.10)	3	17	20	(κ = 0.11)
**Non-responder**	2	32	34		2	32	34	
**Total**	5	49	54		5	49	54	
**MTV**								
**Responder**	4	11	15	(κ = 0.30)	3	11	14	(κ = 0.21)
**Non-responder**	1	38	39		2	38	40	
**Total**	5	49	54		5	49	54	
**TLG**								
**Responder**	3	14	17	(κ = 0.15)	3	14	17	(κ = 0.15)
**Non-responder**	2	35	37		2	35	37	
**Total**	5	49	54		5	49	54	

*Abbreviations*: *CT* Computed tomography, *PET* Positron emission tomography, *RECIST* Response Evaluation Criteria Solid in Tumors, *irRECIST* Immune modified Response Evaluation Criteria Solid in Tumors, *PERCIST* PET Evaluation Criteria in Solid Tumors, *iPERCIST* Immune modified Response Evaluation Criteria Solid in Tumors, *EORTC* European Organization for Research and Treatment of Cancer, *SUL*_*peak*_ The peak standardized uptake value normalized by lean body mass, *MTV* Metabolic tumor volume, *TLG* Total lesion glycolysis, *(κ)* The value of agreement between two assessments using Cohen κ coefficient

**Table 3:** Comparison of therapeutic response evaluation between different PET criteria

Variables	PERCIST				iPERCIST			
	Responder	Non-responder	Total	(κ)	Responder	Non-responder	Total	(κ)
**EORTC criteria**								
**SUL**_**peak**_								
**Responder**	16	4	20	(κ = 0.71)	13	7	20	(κ = 0.62)
**Non-responder**	3	31	34		2	32	34	
**Total**	19	35	54		15	39	54	
**MTV**								
**Responder**	12	2	14	(κ = 0.89)	12	2	14	(κ = 0.89)
**Non-responder**	0	40	40		0	40	40	
**Total**	12	42	54		12	42	54	
**TLG**								
**Responder**	15	2	17	(κ = 0.91)	15	0	15	(κ = 0.91)
**Non-responder**	0	47	47		2	37	39	
**Total**	15	49	54		17	37	54	

*Abbreviations*: *CT* Computed tomography, *PET* Positron emission tomography, *RECIST* Response Evaluation Criteria Solid in Tumors, *irRECIST* Immune modified Response Evaluation Criteria Solid in Tumors, *PERCIST* PET Evaluation Criteria in Solid Tumors, *iPERCIST* Immune modified Response Evaluation Criteria Solid in Tumors, *EORTC* European Organization for Research and Treatment of Cancer, *SUL*_*peak*_ The peak standardized uptake value normalized by lean body mass, *MTV* Metabolic tumor volume, *TLG* Total lesion glycolysis, *(κ)* The value of agreement between two assessments using Cohen κ coefficient

Table 4 shows the comparison of tumor response evaluation between CT and PET criteria according to controlled or uncontrolled diseases. The relationships of PERCIST, iPERCIST, and EORTC with RECIST or irRECIST displayed slight agreement (κ = 0.0 ~ 0.18) (Table 4). In the analysis by MTV, a fair agreement (κ = 0.28 ~ 0.40) was observed among the other criteria, although a poor agreement (κ = 0.0) was observed between iPERCIST and RECIST (Table 4). Analysis by TLG showed a moderate agreement (κ = 0.55 and 0.48) between iPERCIST and RECIST or irRECIST (Table 4). In addition, a comparison of tumor response assessment based on the controlled or uncontrolled disease was performed between the different PET criteria (Table 5). There was a substantial agreement (κ = 0.71) between PERCIST and EORTC and a moderate agreement (κ = 0.41) between iPERCIST and EORTC. The analysis by MTV showed an almost perfect agreement (κ = 0.96) between PERCIST and EORTC and a substantial agreement (κ = 0.74) between iPERCIST and EORTC. The analysis of TLG showed a substantial agreement between (κ = 0.73 and 0.63) EORTC and PERCIST or iPERCIST (Table 5).

**Table 4:** Comparison of therapeutic response evaluation between CT and PET criteria

Variables	RECIST 1.0				irRECIST			
	Controlled	Uncontrolled	Total	(κ)	Controlled	Uncontrolled	Total	(κ)
**PERCIST**								
**SUL**_**peak**_								
**Controlled**	39	9	48	(κ = 0.11)	42	6	48	(κ = 0.18)
**Uncontrolled**	4	2	6		4	2	6	
**Total**	43	11	54		46	8	54	
**MTV**								
**Controlled**	26	0	26	(κ = 0.38)	26	0	26	(κ = 0.28)
**Uncontrolled**	17	11	18		20	8	28	
**Total**	43	11	54		46	8	54	
**TLG**								
**Controlled**	28	1	29	(κ = 0.38)	29	0	29	(κ = 0.33)
**Uncontrolled**	15	10	25		17	8	25	
**Total**	43	11	54		46	8	54	
**iPERCIST**								
**SUL**_**peak**_								
**Controlled**	41	9	50	(κ = 0.18)	44	7	51	(κ = 0.11)
**Uncontrolled**	2	2	4		2	1	3	
**Total**	43	11	54		46	8	54	
**MTV**								
**Controlled**	32	10	42	(κ = 0)	32	0	32	(κ = 0.40)
**Uncontrolled**	11	1	12		14	8	22	
**Total**	43	11	54		46	8	54	
**TLG**								
**Controlled**	34	1	35	(κ = 0.55)	35	0	35	(κ = 0.48)
**Uncontrolled**	9	10	19		11	8	19	
**Total**	43	11	54		46	8	54	
**EORTC criteria**								
**SUL**_**peak**_								
**Controlled**	35	9	44	(κ = 0)	38	6	44	(κ = 0.06)
**Uncontrolled**	8	2	10		8	2	10	
**Total**	43	11	54		46	8	54	
**MTV**								
**Controlled**	27	1	28	(κ = 0.35)	27	0	27	(κ = 0.29)
**Uncontrolled**	16	10	26		19	8	27	
**Total**	43	11	54		46	8	54	
**TLG**								
**Controlled**	31	3	34	(κ = 0.34)	31	3	34	(κ = 0.18)
**Uncontrolled**	12	8	20		15	5	20	
**Total**	43	11	54		46	8	54	

*Abbreviations*: *CT* Computed tomography, *PET* Positron emission tomography, *RECIST* Response Evaluation Criteria Solid in Tumors, *irRECIST* Immune modified Response Evaluation Criteria Solid in Tumors, *PERCIST* PET Evaluation Criteria in Solid Tumors, *iPERCIST* Immune modified Response Evaluation Criteria Solid in Tumors, *EORTC* European Organization for Research and Treatment of Cancer, *SUL*_*peak*_ The peak standardized uptake value normalized by lean body mass, *MTV* Metabolic tumor volume, *TLG* Total lesion glycolysis, *(κ)* The value of agreement between two assessments using Cohen κ coefficient

**Table 5:** Comparison of therapeutic response evaluation between different PET criteria

Variables	PERCIST				iPERCIST			
	Controlled	Uncontrolled	Total	(κ)	Controlled	Uncontrolled	Total	(κ)
**EORTC criteria**								
**SUL**_**peak**_								
**Controlled**	44	0	44	(κ = 0.71)	44	0	44	(κ = 0.41)
**Uncontrolled**	4	6	10		7	3	10	
**Total**	48	6	54		51	3	54	
**MTV**								
**Controlled**	25	1	26	(κ = 0.96)	26	1	27	(κ = 0.74)
**Uncontrolled**	0	28	28		6	21	27	
**Total**	25	29	54		32	22	54	
**TLG**								
**Controlled**	28	6	34	(κ = 0.73)	30	4	34	(κ = 0.63)
**Uncontrolled**	1	19	20		5	15	20	
**Total**	29	25	54		35	19	54	

*Abbreviations*: *CT* Computed tomography, *PET* Positron emission tomography, *RECIST* Response Evaluation Criteria Solid in Tumors, *irRECIST* Immune modified Response Evaluation Criteria Solid in Tumors, *PERCIST* PET Evaluation Criteria in Solid Tumors, *iPERCIST* Immune modified Response Evaluation Criteria Solid in Tumors, *EORTC* European Organization for Research and Treatment of Cancer, *SUL*_*peak*_ The peak standardized uptake value normalized by lean body mass, *MTV* Metabolic tumor volume, *TLG* Total lesion glycolysis, *(κ)* The value of agreement between two assessments using Cohen κ coefficient

### Survival analysis according to different response criteria

As described previously [4], 40 patients experienced disease recurrence, and 21 died due to primary progression. The median PFS and OS were 174 days and not reached, respectively. Table 6 shows the univariate and multivariate survival analyses of different variables, including the tumor response criteria according to CT and PET by SUL_peak_, MTV, and TLG. RECIST and irRECIST as CT criteria were identified as significant predictors of PFS to distinguish controlled from uncontrolled disease, but not OS. Additionally, RECIST and irRECIST could not significantly predict PFS and OS between responders and non-responders. In the SUL_peak_ analysis, iPERCIST, based on responder or non-responder status, was found to be a significant predictor of PFS, as confirmed by multivariate analysis. Analysis by MTV instead of SUL_peak_ demonstrated that PERCIST, iPERCIST, and EORTC according to controlled or uncontrolled disease were significant factors for predicting worse PFS, and multivariate analysis confirmed that PERCIST and EORTC were independent factors for predicting worse PFS. PS, PERCIST, and iPERCIST were significant predictors of OS; however, only PS was confirmed as an independent factor for OS. Moreover, the analysis by TLG demonstrated that PERCIST, iPERCIST, and EORTC based on controlled or uncontrolled disease and EORTC based on responder or non-responder status were significant predictors of PFS and OS. However, the multivariate analysis did not identify any variables as independent predictors.

**Table 6:** Univariate and multivariate survival analysis

Different variables	SUL_**peak**_						MTV						TLG					
	PFS			OS			PFS			OS			PFS			OS		
	UV		MV	UV		MV	UV		MV	UV		MV	UV		MV	UV		MV
	MST	***p***	***p***	MST	***p***	***p***	MST	***p***	***p***	MST	***p***	***p***	MST	***p***	***p***	MST	***p***	***p***
**Age**																		
< 75	168			NR	0.645		168			NR			168			168		
≥ 75	188	0.344		537			188	0.344		537	0.645		188	0.344		188	0.344	
**Sex**																		
Male	174			759			174			759			174			174		
Female	196	0.932		NR	0.499		196	0.932		NR	0.499		196	0.932		196	0.932	
**PS**																		
0–1	239			NR			239			NR			239			239		
2	65	0.079		235	**0.004**	**0.014**	65	0.079		235	**0.004**	**0.016**	65	0.079		65	0.079	
**Histology**																		
AD	178			759			178			759			178			178		
Non-AC	205	0.889		396	0.447		205	0.889		396	0.447		205	0.889		205	0.889	
**RECIST**																		
Res	393			NR			393			NR			393			393		
NRes	174	0.494		759	0.908		174	0.494		759	0.908		174	0.494		174	0.494	
Uncontrol	105			NR			105			NR			105			105		
Control	239	**0.016**	0.632	539	0.807	0.741	239	**0.016**	0.208	539	0.807		239	**0.016**	0.935	239	**0.016**	0.935
**irRECIST**																		
Res	393			NR			393			NR			393			393		
NRes	174	0.494		759	0.908		174	0.494		759	0.908		174	0.494		174	0.494	
Uncontrol	95			NR			95			NR			95			95		
Control	239	**0.001**	0.436	537	0.636		239	**0.001**	0.070	537	0.636		239	**0.001**	0.589	239	**0.001**	0.589
**PERCIST**																		
Res	386			537			393			NR			355			355		
NRes	144	0.066		759	0.954		138	0.105		514	0.136		133	0.119		133	0.119	
Uncontrol	230			NR			108			371			105			105		
Control	178	0.705		537	0.459		566	**< 0.001**	**0.021**	NR	**0.035**	0.770	393	**< 0.001**	0.115	393	**< 0.001**	0.115
**iPERCIST**																		
Res	393			NR			393			NR			355			355		
NRes	133	**0.016**	**0.040**	514	0.288	0.208	138	0.105		514	0.136		133	0.119		133	0.119	
Uncontrol	174			NR			95			371			79			79		
Control	188	0.987		537	0.633		393	**< 0.001**	0.761	NR	**0.030**	0.960	355	**< 0.001**	0.942	355	**< 0.001**	0.942
**EORTC**																		
Res	355			NR			393			NR			393			393		
NRes	165	0.109		759	0.874		129	0.064		514	0.156		125	**0.035**	0.958	125	**0.035**	0.958
Uncontrol	239			NR			111			372			95			95		
Control	168	0.712		759	0.907		393	**0.001**	**0.038**	NR	0.057		355	**< 0.001**	0.749	355	**< 0.001**	0.749

*Abbreviations*: *SUL*_*peak*_ The peak standardized uptake value normalized by lean body mass, *MTV* Metabolic tumor volume, *TLG* Total lesion glycolysis, *PFS* Progression-free survival, *OS* Overall survival, *UV* Univariate analysis, *MV* Multivariate analysis, *MST* Median survival time (days), *p. P*-value, *PS* Performance status, *AD* Adenocarcinoma, *Non-AD* Non-adenocarcinoma, *Res* Responder, *NRes* Non-responder, *NR* Not reached, *Uncontrol* Uncontrolled disease, *Control* Controlled disease, *bold types* Statistically significance

## Discussion

All previous studies focused on tumor response evaluation based on different criteria more than 2 months after the initiation of PD-1 blockade. Response evaluation at the earliest phase should be considered for the continuous administration of immunotherapy. To rule out a non-responder to ICIs, the occurrence of immune-related adverse events and delays in sequential therapy should be considered. In our study, we assessed the potential for early evaluation of tumor response to PD-1 blockade monotherapy based on different response criteria. In the survival analysis according to the response criteria for morphological assessment, PD by RECIST or irRECIST at 4 weeks after PD-1 blockade therapy could significantly predict poor PFS. However, responders at 4 weeks were not identified as significant predictors of outcome. Based on the metabolic assessment, iPERCIST responders at 4 weeks after ICI administration had a significantly better PFS. When MTV or TLG were applied for tumor response assessment, PMD by PERCIST, iPERCIST, or EORTC criteria 1 month after PD-1 blockade was a significant factor for predicting worse outcomes. We found that TLG was used to identify responders and uncontrolled disease 4 weeks after ICI initiation as having significantly better and worse prognoses, respectively, based on EORTC. The results of our study suggest that EORTC by TLG could successfully predict the responder and uncontrolled disease in the early phase after ICI administration compared to the other response criteria. Conversely, PET response criteria using SUV_peak_ was not helpful for predicting survival by responders or uncontrolled disease in the early phase after ICI administration. Considering the concordance rate between the CT and PET response criteria, the agreement between iPERCIST and irRECIST was moderate, showing higher concordance than the other combinations. The agreement for the detection of responders or uncontrolled disease between different PET response criteria was high when using MTV or TLG compared to SUV_peak_. Even if any PET response criteria by MTV or TLG were applied to predict PMD as a significantly worse outcome, all of these criteria seemed to be available in the same way. To predict that the responder has a significantly favorable prognosis, EORTC using TLG may be more helpful than other PET criteria. More research needs to be conducted to evaluate tumor response in the early phase after platinum-based chemotherapy and PD-1 blockade.

Recently, Ayati et al. retrospectively performed a tumor response assessment according to RECIST, irRECIST, PERCIST, and iPERCIST after a median of four cycles of PD-1 blockade monotherapy in 72 patients with advanced NSCLC [14]. The results of their study showed that most ^18^F-FDG accumulation lesions on PERCIST and iPERCIST accurately reflected the overall metabolic response [14]. However, all studies using PET response criteria for immunotherapy focused on approximately 2 months after its initiation, and the ability to predict survival was slightly different according to individual studies [11, 13–15]. In a comparison of CT and PET criteria for assessing response to PD-1 blockade, RECIST and PERCIST were found to have a moderate agreement (κ = 0.41-0.60) [11]. In the present study, we explored the prospective data of advanced NSCLC patients who underwent ^18^F-FDG PET at 4 and 9 weeks after the initiation of PD-1 blockade monotherapy and compared the concordance of tumor response according to CT and PET response criteria in the early phase after the beginning of ICIs with the outcome. Our previous approach indicated that PERCIST by MTV or TLG was superior to RECIST for predicting therapeutic response and survival 4 weeks after PD-1 blockade [4]. However, the PERCIST defined by SUL_peak_ exhibited an inferior therapeutic predictivity to that of MTV or TLG and could not accurately discriminate controlled from uncontrolled disease 4 weeks after PD-1 blockade [4]. Early detection of the efficacy of PD-1 blockade prevents the delay of sequential therapy and the deterioration of the general condition. In general, morphological changes on CT can successfully detect tumor response 9 weeks after PD-1 blockade initiation. If not in the early phases of ICI treatment, response assessment using PET criteria instead of a CT scan may be unnecessary. In our study, the PET criteria (PERCIST, iPERCIST, and EORTC) defined not by SUL_peak_ but by MTV or TLG were useful for predicting worse outcomes in patients with uncontrolled disease in the early phase. In particular, the EORTC defined by TLG was identified as a significant marker for predicting the outcome of uncontrolled disease and responders in the early phase. Thus, we would like to suggest that not SUL_peak_ but MTV or TLG is suitable when defining the PET criteria for tumor response assessment, and the EORTC criteria defined by TLG are effective for accurately predicting the outcome according to different tumor responses when assessed at an early phase, such as 1 month after ICI administration. A large sample size is required to confirm the usefulness of the PET response criteria for MTV or TLG.

Taken together, irRECIST and iPERCIST yielded an advantage for predicting the poor outcome of the patients with uncontrolled disease at early phase after ICI administration, however, there was weak merit to predict the favorable prognosis for the patients with responder. On the other hand, EORTC was useful for predicting the outcome in the patients presenting both uncontrolled disease and responder at early phase after ICI administration. irRECIST provided a consistent result regardless of different metabolic parameters such as SUL_max_, MTV or TLG. However, iPERCIST and EORTC were identified as useful criteria by ^18^F-FDG accumulation based on MTV or TLG compared to that on SUL_max_.

The current study had several limitations. First, our study was limited to an Asian population with a very small sample size and included a widely varied patient population, which may have biased the results. A larger sample size is necessary to elucidate the therapeutic significance of tumor response assessment. Second, the current study included a heterogeneous population of patients with NSCLC who received first-line or second-line treatments, or more PD-1 blockades. Currently, ICI treatment is identified as the standard first-line treatment. Further investigation is warranted to focus on the assessment of the tumor response to first-line PD-1 blockade. Finally, combination therapy, such as nivolumab plus ipilimumab or platinum-based chemotherapy plus pembrolizumab, is usually administered to patients with advanced NSCLC. CT and PET criteria should be used to examine the therapeutic significance of tumor response assessment in the early phase after the initiation of combined immunotherapy.

## Conclusion

EORTC criteria based on TLG for the early detection of responders or uncontrolled disease were useful for response assessment after PD-1 blockade therapy. Although RECIST or irRECIST may be possible for detecting uncontrolled disease 4 weeks after ICI treatment, irRECIST yielded a moderate agreement with iPERCIST by TLG. When MTV or TLG instead of SUL_peak_ was used for the PET response criteria, the agreement between EORTC and PERCIST or iPERCIST was almost perfect. The prognostic significance of PET response assessment between 4 weeks and more than 2 months after ICI initiation may be different. Further studies should focus on tumor response assessment as early as possible after ICI administration to accurately distinguish responders from uncontrolled disease.

## Supplementary Information

**Additional file 1: Table A1.** Comparison of response evaluation between RECIST and iRECIST.

**Additional file 2: Table A2.** Comparison of response evaluation between PERCIST and iPERCIST.

## Data Availability

All data generated or analyzed during this study are included in this published article. The datasets used and/or analysed during the current study available from the corresponding author on reasonable request.

## Abbreviations

ICIs: Immune checkpoint inhibitors
PD-1: Programmed death-1
PD-L1: Programmed death-ligand-1
NSCLC: Non-small cell lung cancer
^18^F-FDG: 2-deoxy-2-[fluorine-18]-fluoro-d-glucose
PET: Positron emission tomography
CT: Computed tomography
PERCIST: PET Response Criteria in Solid Tumors
RECIST: Response Evaluation Criteria in Solid Tumors
irRECIST: Immune-modified RECIST
iPERCIST: Immune-modified PERCIST
EORTC: Organization for Research and Treatment of Cancer
SUV: Standardized uptake value
LBM: Lean body mass
SUL: SUV corrected for LBM
VOI: Volume of interest
SUL_max_: Maximum SUL
SUL_peak_: Peak SUV normalized by LBM
MTV: Metabolic tumor volume
TLG: Total lesion glycolysis
CR: Complete response
PR: Partial response
SD: Stable disease
PD: Progressive disease
CMR: Complete metabolic response
PMR: Partial metabolic response
SMD: Stable metabolic disease
PMD: Progressive metabolic disease
PFS: Progression-free survival
OS: Overall survival
PS: Performance status
