# Supplementary material for: Comparative analysis of different response criteria at early phase after PD-1 blockade in non-small lung cancer
Source: Cancer Imaging. 2023 Mar 1;23:23. doi: 10.1186/s40644-023-00538-x (PMC9976499; doi:10.1186/s40644-023-00538-x)
Supplement: Supplementary file 1 — Additional file 1: Table A1. Comparison of response evaluation between RECIST and iRECIST. [file 40644_2023_538_MOESM1_ESM.docx]

**Table A1. Comparison of response evaluation between RECIST and iRECIST**

|  | RECIST 1.0 | | |
| --- | --- | --- | --- |
| **irRECIST** | Responder | Non-responder | Total |
| Responder | 5 | 0 | 5 |
| Non-responder | 0 | 49 | 49 |
| Total　κ=1.0 | 5 | 49 | 54 |
