# Supplementary material for: Comparative analysis of different response criteria at early phase after PD-1 blockade in non-small lung cancer
Source: Cancer Imaging. 2023 Mar 1;23:23. doi: 10.1186/s40644-023-00538-x (PMC9976499; doi:10.1186/s40644-023-00538-x)
Supplement: Supplementary file 2 — Additional file 2: Table A2. Comparison of response evaluation between PERCIST and iPERCIST. [file 40644_2023_538_MOESM2_ESM.docx]

**Table A2. Comparison of response evaluation between PERCIST and iPERCIST**

| iPERCIST | PERCIST | | |
| --- | --- | --- | --- |
| **SUL_peak_** | Responder | Non-responder | Total |
| Responder | 14 | 1 | 15 |
| Non-responder | 5 | 34 | 39 |
| Total　κ=0.74 | 19 | 35 | 54 |
|  | | | |
| **MTV** | Responder | Non-responder | Total |
| Responder | 12 | 0 | 12 |
| Non-responder | 0 | 42 | 42 |
| Total κ=1.0 | 12 | 42 | 54 |
|  | | | |
| **TLG** | Responder | Non-responder | Total |
| Responder | 15 | 0 | 15 |
| Non-responder | 0 | 49 | 49 |
| Total κ=1.0 | 15 | 49 | 54 |
